# Extracting the Evaluations of Stereotypes: Bi-factor Model of the Stereotype Content Structure

**DOI:** 10.3389/fpsyg.2017.01692

**Published:** 2017-10-04

**Authors:** Pablo Sayans-Jiménez, Isabel Cuadrado, Antonio J. Rojas, Juan R. Barrada

**Affiliations:** ^1^Department of Psychology, Faculty of Psychology, University of Almería, Almería, Spain; ^2^Center for the Study of Migration and Intercultural Relations, University of Almería, Almería, Spain; ^3^Department of Psychology and Sociology, Faculty of Social Sciences and Humanities, University of Zaragoza, Teruel, Spain

**Keywords:** stereotype content, global evaluation, intergroup attitudes, intergroup evaluations, bi-factor model

## Abstract

Stereotype dimensions—competence, morality and sociability—are fundamental to studying the perception of other groups. These dimensions have shown moderate/high positive correlations with each other that do not reflect the theoretical expectations. The explanation for this (e.g., halo effect) undervalues the utility of the shared variance identified. In contrast, in this work we propose that this common variance could represent the global evaluation of the perceived group. Bi-factor models are proposed to improve the internal structure and to take advantage of the information representing the shared variance among dimensions. Bi-factor models were compared with first order models and other alternative models in three large samples (300–309 participants). The relationships among the global and specific bi-factor dimensions with a global evaluation dimension (measured through a semantic differential) were estimated. The results support the use of bi-factor models rather than first order models (and other alternative models). Bi-factor models also show a greater utility to directly and more easily explore the stereotype content including its evaluative content.

## Introduction

Stereotypes are the attributes considered characteristic of social groups, or of the people belonging to them (Stangor, 2009). They are an essential element for studying intergroup attitudes and evaluations. By virtue of stereotype content, it is possible to know what types of behaviors or results are expected from interaction with the group or the people evaluated. Specifically, the most relevant stereotype content is the one that informs about a group’s (or its members’) competence and warmth (Fiske et al., 1999, 2002). *Competence* dimension represents the characteristics related to the efficiency of the social object to achieve his/her goals (e.g., clever, creative, efficient, and intelligent), whereas *warmth* refers to the benevolence of these goals (e.g., good-natured, trustworthy, tolerant, friendly, and sincere; Goodwin et al., 2014).

Recent studies on the formation of impressions of people or groups reveal that there are, in fact, two components to the warmth dimension—morality and sociability—and, of the two, morality has been shown to exercise a more dominant influence (Leach et al., 2007; Brambilla et al., 2011, 2012; Brambilla and Leach, 2014; Goodwin et al., 2014). *Morality* dimension accounts for the moral goals of the social object, as well as the damages and benefits that the social object may produce in terms of damage or care/welfare (e.g., honest, sincere, trustworthy). Meanwhile, *sociability* is defined by the goal of cooperation, reciprocity, and/or the bonds created with other people or groups (e.g., open, friendly, likeable; Goodwin et al., 2014). Currently, both proposals (two and three dimensions of the stereotype content) coexist and are accepted as valid for the study of intergroup attitudes. This study will adopt the proposal with three dimensions because we consider it as a more exhaustive representation for the stereotype content.

### The Measurement of Stereotype Content

**FIGURE 1:**
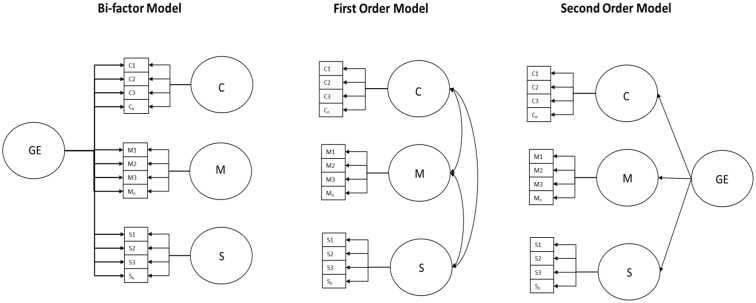
Bi-factor, first and second order measurement models. GE, Global Evaluation; C, Competence; M, Morality; S, Sociability.

Although the use of stereotype content has taken root in the field of intergroup relations there are still possibilities for improving its measurement. In particular, this study claims that bi-factor models (BMs) are the best and the most useful way to interpret the structure of people’s answers to self-reported perceptions of other groups using the stereotype content. Conventionally, first order models (FOMs) are used to explore stereotype content (see **Figure 1**; Fiske et al., 1999; Eckes, 2002; Lin et al., 2005; López-Rodríguez et al., 2013). However, these models cannot explain theoretically (nor use) the large amount of shared variance among the items of the competence, morality, and sociability dimensions (or competence and warmth dimension subsuming morality and sociability). Therefore, the objective of this work is to demonstrate that, when the groups are perceived and their elicited stereotype content is measured with self-report techniques, the universal dimensions of social perception (i.e., competence, morality, and sociability) share a big amount of variance regarding the connotative evaluation of the perceived group. The fulfillment of the previous assumption highlights two major problems related to the measurement of the stereotype content elicited by a perceived group: (1) there exist validity problems with respect to the internal structure when self-report scores of stereotype content scales are interpreted, and (2) a lot of useful information regarding the evaluation of the social object is being wasted. To achieve the stated objective BMs will be compared to FOMs currently used (with competence, sociability, and morality dimensions), and with alternative models such as those with one single factor (SFMs; in which the latent construct would be only a global evaluation) or second order factor models. Additionally validity evidences based on relationships with measures of other variables will be collected in order to test if the BM common dimension can be interpreted as a global evaluation of the perceived outgroup.

One of the most common techniques utilized to explore stereotypes and their content is self-report. This technique permits the use of large sets of items, reducing the random error, and allowing access to empirical evidence based on large samples and multiple constructs (Krosnick et al., 2005). However, the studies that employ competence, morality, and sociability dimensions (or competence and warmth dimension subsuming morality and sociability) have found moderate/high empirical correlations among these dimensions that do not correspond to their theoretical definitions (Fiske et al., 1999; Eckes, 2002; Judd et al., 2005; Lin et al., 2005; López-Rodríguez et al., 2013; Sayans-Jiménez et al., 2017). In this regard, the assessment of the social object’s ability to perform a behavior (i.e., competence) should not be related to the positivity or negativity of his or her goals. Therefore, it necessarily follows that there must be an alternative explanation for the shared variance among these dimensions.

### High Correlations between Stereotype Dimensions

Judd et al. (2005) posit that the positive moderate/high correlations between competence and warmth (dimension subsuming morality and sociability) obtained in numerous studies are evidence of a halo effect (the perception that certain characteristics are influenced by other characteristics). On the other hand, these authors claim that there could be negative correlations between competence and warmth, but that these types of correlations would take place only in situations where two groups are compared (rather than where one or several groups are being evaluated without a supervening comparison) and would, furthermore, be generated by a compensatory comparative process.

The high positive correlations between the dimensions can be interpreted as an indicator of a shared relationship among all the stereotypes employed to assess the content of competence, morality, and sociability. This same effect has been detected in the basic dimensions for interpersonal perception (i.e., the Big Five factors or agency and communion; see Srivastava et al., 2010). Far from considering these correlations as a systematic error of raters (e.g., acquiescence) that has to be eliminated, we consider that the information reflected by the shared variance of all indicators could be of great relevance to predicting intergroup evaluations.

Recent attitude models such as the iterative reprocessing model (Cunningham and Zelazo, 2007; Cunningham et al., 2007; Van Bavel et al., 2012b) offer an explanation for the relation found between all the stereotypes that people employ to describe attitude targets. The iterative reprocessing model posits that the evaluations shown by the raters are influenced by both evaluative-typified (offering valence and arousal) and reflective-typified processes (integrating information of the evaluative processes, the context, the goals of the perceiver, and the additional information of the target; Cunningham et al., 2004, 2007). There is also evidence that people, besides expressing their evaluation using the stereotype content, evaluate social targets along a general *good*–*bad* dimension (Fazio et al., 1986; Bargh et al., 1992; Cunningham and Van Bavel, 2009). In the case of interpersonal perception, Srivastava et al. (2010) proposed to capture this global evaluation by applying a multidimensional structure with a second order global evaluation factor and trait specific factors. However, in the present study it is assumed that there are enough theoretical considerations to capture the variance (shared among stereotype items) due to the global evaluation using BMs.

Bi-factor models will establish that the relation among all the items of the stereotype content dimensions is only due to the shared global evaluation of the social target and not to their specific meaning regarding competence, morality, and sociability (**Figure 1**). However, in the case of second order factor models the relation among items is due to the bonds between competence, morality, and sociability dimensions, which does not correspond with the theoretical of dimensions nor to the proposed use of the scores. The assessment of the competence, morality, and sociability content (or competence and warmth dimension subsuming morality and sociability) seeks to predict specific emotional reactions linked to concrete behavioral tendencies. To that end, it is necessary to know the position of the groups (or people belonging to them) in each stereotype dimension, which supposedly have to vary independently. Therefore, for these models it is imperative to access the specific content of each dimension (as opposed to the content shared among dimensions). This is the soundest reason for using BMs instead of second order models (Murray and Johnson, 2013).

Bi-factor models (**Figure 1**) summarize the relationship between the employed items in two types of factors: one general, shared among all the items (in the case of stereotypes this factor would represent the global evaluation), and another specifically composed of the groups of items that reflect the specific content of each dimension (Brunner et al., 2012), in this case competence, morality, and sociability. On the other hand, FOM items are influenced only by a specific factor depending on the content dimension (competence, morality, or sociability).

We assume that the answers to stereotype scales when a group is perceived will reflect two differentiated sources of variance—the first relating to the global evaluation (positive/negative), which is influenced by the connotative value of the target’s social category belonging; and, the second concerning a more nuanced evaluation derived from reflexive information. These evaluations would constitute the specific factors (i.e., competence, morality, and sociability) after considering the effect of the global factor.

Belonging to specific social categories carries strong evaluative connotations (Van Bavel and Cunningham, 2009). However, the effect of this global evaluation (positive/negative) has never been isolated from the competence, morality, and sociability content (or competence and warmth dimension subsuming morality and sociability). The effect of the information coming from the global evaluation in the processing of the entire evaluative system would be an explanation for the moderate/high positive correlations when a group (or the people belonging to it) is evaluated. In other words, when people try to describe the most salient characteristics of a social object using the stereotype content (i.e., competence, morality, and sociability) they also reflect the global evaluation of the social object.

### The Present Research

To fulfill our aim, BMs are considered as the best way to interpret the structure of people’s answers to stereotype self-reports when they represent the perception of other groups. To test the BMs’ feasibility, three samples with different targets were used. One such sample answered questions regarding the stereotypes of *people of the gypsy ethnic group* (Sample_G_), another sample addressed issues with respect to the stereotypes of *professional firefighters* (Sample_F_), and the third sample dealt with questions in relation to the stereotypes of *people with Down syndrome* (Sample_D_). These groups were selected to test BMs in the context of two related aims: contrasting a highly valued outgroup (professional firefighters) in the competence, morality, and sociability dimensions with a lowly valued outgroup (gypsy ethnic group from which lower or more negative scores were expected)^1^; and, secondly, evaluating a so-called ambivalent group, people with Down syndrome, from whom low competence and high morality and sociability were expected (Fiske et al., 2002). The choice of these three group types was intended to facilitate the generalization of the results of the study as it will now be possible to test the structure of answers with three different patterns of response from the dimensions of competence, morality, and sociability.

Although the theoretical reasons point to choosing BMs instead of FOMs (e.g., the need to access the pure content of the dimensions of competence, morality, and sociability), other alternative models—i.e., SFMs and second order factor models where the common dimension represent a global evaluation—were also tested. Finally, in order to test that the common factor among stereotype items reflects a global evaluation of the outgroup target (instead of a systematic error of the raters such as acquiescence) the relation between the common factor and a measurement of the outgroup global evaluation using a semantic differential of evaluation (SDE) was estimated. The SDE is a common method to assess positive/negative global evaluation (Osgood et al., 1957; Díaz-Guerrero and Salas, 1975) through the relation between the items’ and the target’s connotative evaluative content. Positive moderate/high correlations were expected between the SDE and the BMs’ global evaluation in the three samples. Our expectations point to a better fit of the BMs in contrast to FOMs or SFMs. Furthermore, we expect moderate/high relationship supporting the interpretation of the BM common dimension as the global evaluation of the perceived group.

## Materials and Methods

### Participants

Nine hundred and nine people in the general population, divided into three samples of 300, 300, and 309 people, participated in the study (none of them belonged to the gypsy ethnic group, were professional firefighters or were people with Down syndrome). They were mainly recruited at their place of residence with random routes, by quota sampling, in two towns of Spain. The gender quota was 50% for women and 50% for men. The ranges for age quota were 30–32% for ages between 18- and 35-years-old, 38% for ages between 36- and 55-years-old, and 30–32% for 56 and older. One sample was asked about attitude toward the gypsy ethnic people (Sample_G_), another sample, about attitude toward professional firefighters (Sample_F_), and the third sample was asked about attitude toward people with Down syndrome (Sample_D_).

### Instruments

Three instruments were administered: a questionnaire including competence, morality, and sociability scales; SDE; and some socio-demographic data items (i.e., sex, age). Each sample had a different outgroup target (gypsy ethnic people, professional firefighters, and people with Down syndrome). The scales were applied in Spanish.

#### Competence, Morality, and Sociability Scales

Each scale measured how “non-gypsy people,” “non-firefighters,” and “non-Down syndrome” people represented the targets according to competence, morality, and sociability dimensions (the items can be seen in **Table 1**). Seven point Likert scales were used (*none, almost none, few, half, many, almost all*, and *all*, the item scores were ranged from 1 to 7). The higher the value of the answer, the greater the perceived association between the target and the trait. Items in the three dimensions were all presented jointly in random order following the same instructions (Appendix 1). The items used were the same (also in Spanish language) than those applied in the positive scales of Sayans-Jiménez et al. (2017). Negative items of this study were omitted because the aim of this study is to test the internal structural alternatives to the most common way to measure the stereotype content (i.e., using only positive items; e.g., Fiske et al., 2002; Brambilla et al., 2011). The purpose of the instructions was that the person who would answer the scale would not focus on specific individual characteristics of the outgroup people that they might know personally (no information on the individual identity of the members of the group was gleaned).

**Table 1:** Descriptive statistics for all the items in the three samples (Sample_G_, Sample_F_, and Sample_D_).

	Sample_G_			Sample_F_			Sample_D_		
			
	*M (SD)*	*K*	*Sk*	*M (SD)*	*K*	*Sk*	*M (SD)*	*K*	*Sk*
**Competence**									
Skillful [Habilidosas]	4.09 (1.05)	0.17	0.52	5.01 (1.08)	-0.06	0.03	3.82 (1.05)	-0.01	0.31
Intelligent [Inteligentes]	3.99 (1.26)	-0.20	-0.15	4.43 (1.10)	0.10	0.17	4.00 (1.32)	-0.10	0.11
Capable [Capacitadas]	4.32 (1.12)	-0.21	0.16	5.79 (1.02)	-0.32	-0.90	4.09 (1.28)	-0.16	0.18
Creative [Creativas]	3.88 (1.25)	0.19	-0.12	6.03 (0.98)	-0.79	-0.11	4.06 (1.21)	-0.30	0.18
**Morality**									
Respectful [Respetuosas]	3.31 (1.23)	0.19	0.17	4.95 (1.22)	-0.03	-0.25	5.07 (1.07)	-0.41	-0.03
Sincere [Sinceras]	3.52 (1.21)	0.14	0.19	5.32 (1.12)	-0.30	-0.36	5.30 (1.18)	-0.18	-0.50
Honest [Honestas]	3.73 (1.22)	0.01	0.12	5.61 (0.95)	-0.15	-0.68	5.28 (1.15)	-0.65	-0.24
Trustworthy [De Confianza]	2.94 (1.26)	0.35	0.21	5.19 (1.26)	-0.49	-0.04	4.68 (1.35)	-0.12	-0.39
Reliable [Formales]	3.31 (1.23)	0.30	0.25	5.58 (1.06)	-0.41	0.06	4.74 (1.14)	-0.10	-0.08
**Sociability**									
Likeable [Simpáticas]	4.65 (1.05)	-0.44	0.60	4.86 (0.97)	0.27	-0.33	5.27 (1.09)	0.05	-0.44
Affectionate [Cariñosas]	4.35 (1.13)	-0.48	0.51	4.80 (1.06)	0.16	-0.03	5.57 (1.00)	1.22	-0.91
Open [Abiertas]	4.74 (1.35)	-0.60	0.19	5.09 (1.04)	0.21	-0.60	4.77 (1.25)	-0.53	-0.07
Friendly [Amistosas]	4.35 (1.18)	-0.56	0.60	4.99 (0.99)	0.08	-0.32	5.44 (0.98)	0.01	-0.29
**Semantic differential**									
Bitter–Sweet [Amargas–Dulces]	3.88 (1.27)	-0.01	-0.31	4.94 (0.97)	0.06	-0.10	4.74 (1.34)	-0.67	-0.18
Opaque–Transparent [Opacas–Transparentes]	3.48 (1.57)	0.51	-0.64	5.15 (1.22)	-0.36	-0.31	4.02 (1.20)	-0.38	0.17
Dark–Light [Oscuras–Claras]	3.49 (1.41)	0.56	-0.29	5.33 (1.04)	-0.48	-0.29	5.00 (1.21)	-0.16	-0.40
Imperfect–Perfect [Imperfectas–Perfectas]	3.40 (1.18)	0.25	0.06	4.91 (1.17)	-0.36	-0.01	5.37 (1.29)	-0.11	-0.71
Broken–Whole [Rotas–Enteras]	4.38 (1.57)	-0.14	-0.98	5.62 (1.26)	-0.83	0.26	5.46 (1.23)	-0.18	-0.74
Unpleasant–Tasty [Desagradables–Sabrosas]	3.86 (1.27)	0.22	-0.17	4.96 (0.96)	-0.19	0.69	5.61 (1.18)	0.16	-0.72
Poisonous–Innocuous [Venenosas–Inocuas]	3.67 (1.33)	0.27	-0.52	4.94 (0.97)	-0.81	0.70	5.51 (1.37)	1.07	-1.13


**M*, Mean; *SD*, Standard Deviation; *K*, Kurtosis; Sk, Skewness.*

#### Semantic Differential

A seven-item SDE with a seven-point response scale validated in Spanish (Díaz-Guerrero and Salas, 1975; Sayans-Jiménez et al., 2017) was used. All the items referred to the evaluation dimension. The pairs of adjectives used were: Sweet–Bitter, Transparent–Opaque, Light–Dark, Perfect–Imperfect, Whole–Broken, Tasty–Unpleasant, and Innocuous–Poisonous (the item scores were ranged from 1 to 7). The order and the direction of the items were randomized to control method effects (acquiescence and item wording effects—positive/negative). (The instructions for the SDE may be found in the Appendix 1). After the application, the items were recoded so that they could be interpreted more easily. Higher scores implied more positive evaluations.

### Procedure

The survey was administered by trained staff. All the questionnaires were administered in different places and times (i.e., mainly at participants’ place of residence). There was no time limit. Respondents’ anonymity and confidentiality were guaranteed. The trained staff read out loud a text specifying that all the data would be handled anonymously and in a global and statistic way. This text also indicated that the participation was voluntary and that it could be stopped at any time. Every participant was informed that they could obtain a copy of their answers. Furthermore, the contact details of the ultimately responsible of the study were provided. Finally, the trained staff confirmed that all the participants were over 18, that they were participating voluntarily, that they knew their answers would be anonymous and will be handled with scientific purposes, that they were aware they could stop their collaboration at any time, and that they were participating freely. This procedure was approved by the Human Research Bioethical Committee of University of Almería, Spain. All subjects gave written informed consent in accordance with the Declaration of Helsinki. The protocol was approved by the Human Research Bioethical Committee.

### Data Analysis

The descriptive statistics of all the indicators were analyzed. Confirmatory factor analysis (CFA) approaches were used to test the relationships among different constructs, reliability, and the adequacy of the factorial structure of the three scales. Covariance matrix was analyzed. The Maximum Likelihood (ML) method was used to estimate the parameters. Analyses were performed using SPSS v19.0 (IBM Corp, 2010) and lavaan (Rosseel, 2012) and seemTools (semTools Contributors, 2016) R packages. Listwise deletion was used to deal with the missing data in Sample_D_. The latent factors metric was assigned by fixing the first loading to 1.00 for all the latent variables except in the structural models where the factor metric was assigned by fixing latent variances to one. In the FOMs, all the correlations between factors were freed, whereas in the BMs the correlations among all factors were fixed to zero. The dataset contained 300 complete cases in Sample_G_ and Sample_F_, whereas Sample_D_ had 299 complete cases. The complete dataset can be found at Sayans-Jiménez (2017).

#### Internal Structure Analysis

Three kinds of transversal factor models were tested in the three samples^2^. The first one, FOM, is that which is commonly used to explore the content of the three stereotype dimensions^3^. The second model, BM, established that, besides the factor related to the specific content of competence, morality, and sociability, there was a common factor shared among all the items that, due to the global evaluations of the outgroup targets, could account for the variance^4^. Finally, a model with one single factor (SFM), representing a global evaluation, was specified. Fit to the models was checked using the chi-square test, the Tucker-Lewis Index (TLI), the comparative fit index (CFI) and the root mean square error approximation (RMSEA) with its 90% confidence interval (90% CI). Fit indices are considered good when RMSEA ≤ 0.05 or CFI ≥ 0.97 (Schermelleh-Engel et al., 2003); adequate when RMSEA is close to 0.06 or CFI ≥ 0.95 (Hu and Bentler, 1999). RMSEA values between 0.08 and 0.10 and CFI values between 0.95 and 0.90 are considered as an acceptable fit. RMSEA values higher than or equal to 0.10 and CFI values lower than 0.90 indicate that the model should be discarded (Brown, 2006).

Because BMs and FOMs are nested within SFMs, chi-square and CFI differences test were performed to guide the choice of the model in the three samples. CFI differences lower than 0.01 indicate that the models have practically no significant differences in fit (Cheung and Rensvold, 2002). If practically significant differences are found, it can be assumed that BMs are able to successfully capture the correlations among first order factors.

#### Estimation of Reliability

Cronbach’s alpha, omega, and hierarchical omega coefficients were estimated (McDonald, 1999; Zinbarg et al., 2005, 2006). Omega estimation for each dimension accounted for all the common variance of their items (i.e., the variance was due to both specific and global factors). Therefore, omega estimations were the same for the FOMs and the BMs in each dimension (i.e., competence, morality, and sociability). Hierarchical omega estimations were under the influence of only the relation between the items and the correspondent factor (the global evaluation, or competence, morality, or sociability). In this regard, adequate omega (and hierarchical omega) values should be higher than 0.80 (Raykov and Marcoulides, 2011). In hierarchical omega estimation it was expected that the proportion of variance due only to the specific content of each dimension would be lower in the specific dimensions (i.e., competence, morality, and sociability) than in the common dimension (i.e., global evaluation).

#### Evidence of Validity Based on Relationships with Measures of Other Variables

To test whether the common factor among all the items corresponded to a global evaluation, an additional measurement was carried out using a SDE on the same outgroup target. The relationship between the SDE and all the modeled stereotype dimensions (i.e., competence, morality, sociability, and global evaluation) was estimated in BMs in the three samples. The data set contained 300 complete cases in Sample_G_ and Sample_F_, whereas Sample_D_ had 287 complete cases.

## Results

The descriptive statistics were calculated for all the items (**Table 1**). No item showed extreme skewness or kurtosis. A simple sight to raw scores (**Table 1**) made it possible to confirm the three patterns of response associated with the outgroup features. In general, the group of professional firefighters were highly valuated in the three dimensions, the group of people with Down syndrome (the ambivalent group) obtained high associations with morality and sociability and lower associations with competence. Finally, the gypsy ethnic group obtained lower scores in the three dimensions.

### Internal Structure Analysis

Fit statistics can be seen in **Table 2**. The chi-squared test showed lack of fit with data in all models. According to other less restrictive indicators, SFMs (with only one latent construct representing global evaluation) should be definitively discarded as possible internal structure in the three samples (henceforth the analysis continued excluding this model). However, BMs and FOMs models showed adequate/good fit in Sample_G_ and in Sample_F_, whereas in Sample_D_ fit indicators were acceptable. Chi-square and CFI differences favoring BMs (in contrast to FOMs) were found in the three samples (**Table 2**). In addition, it should be noted that the possible second order factor model of competence, morality, and sociability was statistically equivalent (Brown, 2006) to the correlated three-factor model shown in FOMs. This made it possible to state that BMs would also show favorable Chi-square and CFI differences if they were compared to a second order factor model of competence, morality, and sociability dimensions.

**Table 2:** Fit statistics, in all the samples, for single factor model (SFM), first order model with three subdimensions (FOM), bi-factor model with three subdimensions (BM), and BM related with semantic differential of evaluation (BM and SDE).

		χ^2^	Δχ^2^	*df*	-Δ*df*	RMSEA [90% CI]	CFI	ΔCFI	TLI
**Model comparison**									
Sample_G_	SFM	437.52	319.44**	65	3	0.14 [0.13, 0.15]	0.79	-0.18	0.75
	FOM	118.08	0.00	62		0.06 [0.04, 0.07]	0.97	0.00	0.96
	BM	91.00	-27.08*	52	-10	0.05 [0.03, 0.07]	0.98	0.01	0.97
Sample_F_	SFM	248.78	80.67**	65	3	0.10 [0.08, 0.11]	0.90	-0.04	0.88
	FOM	168.11	0.00	62		0.08 [0.06, 0.09]	0.94	0.00	0.93
	BM	125.27	-42.84**	52	-10	0.07 [0.05, 0.08]	0.96	0.02	0.94
Sample_D_	SFM	733.34	511.58**	65	3	0.18 [0.17, 0.19]	0.67	-0.25	0.60
	FOM	221.76	0.00	62		0.08 [0.08, 0.10]	0.92	0.00	0.91
	BM	150.66	-71.1**	52	-10	0.08 [0.06, 0.09]	0.95	0.03	0.93
**Structural models**									
Sample_G_	BM and SDE	290.80		153		0.06 [0.05, 0.06]	0.95		0.93
Sample_F_	BM and SDE	317.35		153		0.06 [0.05, 0.07]	0.93		0.91
Sample_D_	BM and SDE	395.29		153		0.07 [0.06, 0.08]	0.91		0.89


*χ^*2*^, Chi-square test; Δχ^*2*^, statistically significant Chi-square differences at *p* < 0.001 by sample between the model and the FOM; *df*, Degrees of freedom; Δ*df*, Degrees of freedom differences by sample between the model and the FOM; RMSEA, Root Mean Square Error of Approximation; 90% CI, 90% Confidence Interval of the RMSEA; CFI, Comparative Fit Index; ΔCFI, CFI differences by sample between the model and the FOM; TLI, Tucker–Lewis Index. ^∗^*p* < 0.01, ^∗∗^*p* < 0.001.*

**FIGURE 2:**
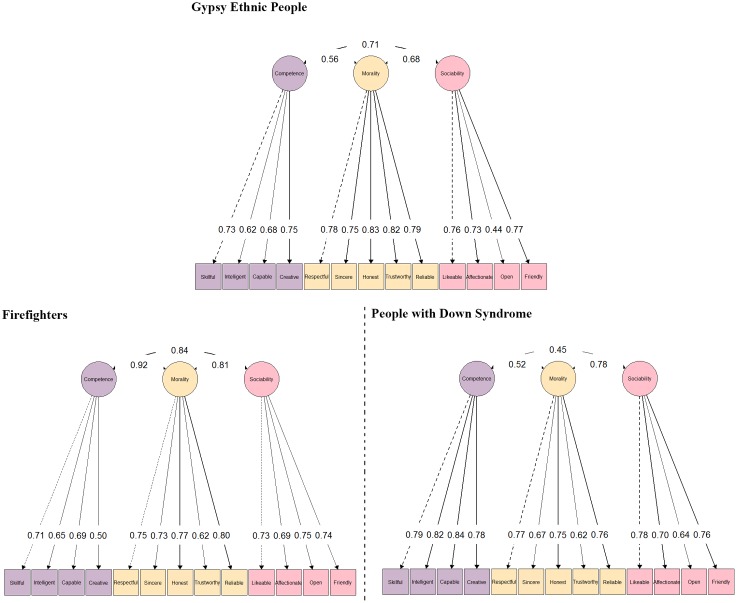
Standardized factor loadings and correlations in first order factor model with three subdimensions (FOM) in the three samples. Latent variable colors determine, based on their relationship, the color of the manifest variables. All factor loadings are statistically significant.

**FIGURE 3:**
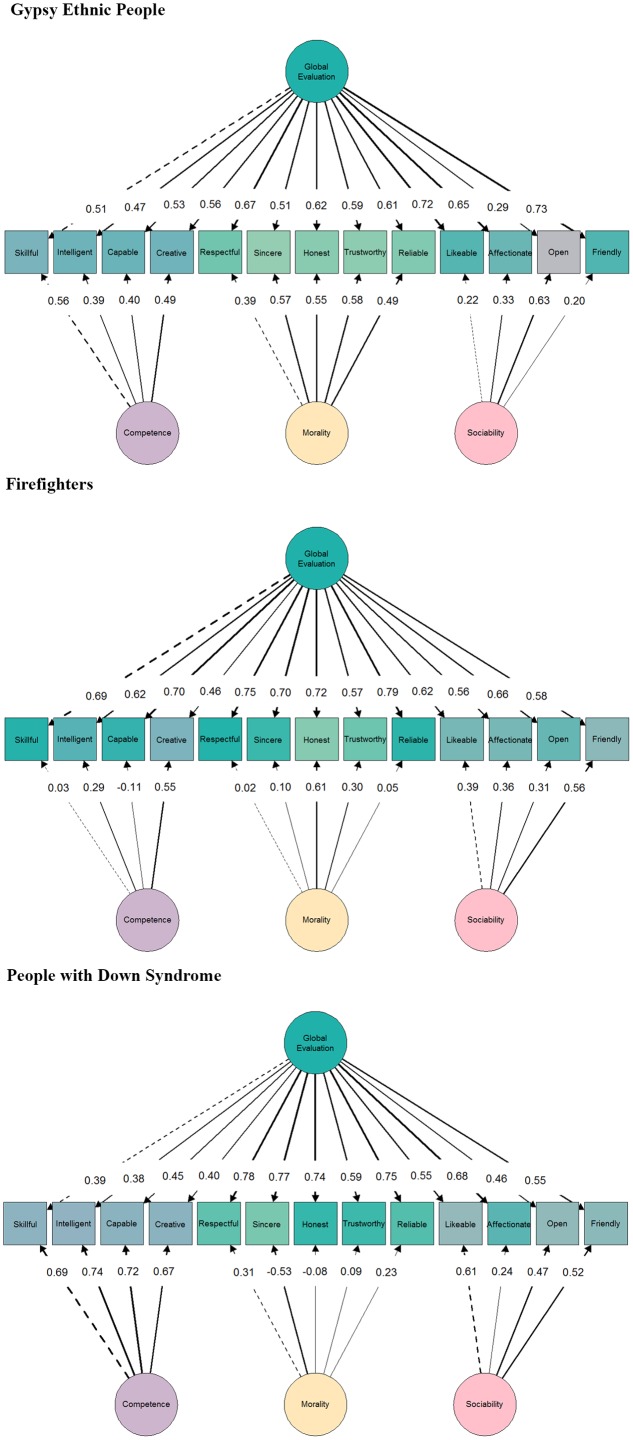
Standardized factor loadings in Bi-factor model with three subdimensions (BM) in the three samples. Latent variable colors determine, based on their relationship, the color of the manifest variables. Dashed edges represent *a priori* fixed factor loading. All factor loadings on the global factor are statistically significant. In the case of the specific factors, items loadings for open in Sample_G_, all the Competence and Morality items in Sample_B_ (except those used for assigning the metric), and honest, trustworthy and reliable in Sample_D_ are not statistically significant.

From the results shown in the three samples, we consider that the model with the overall better fit was BM. Therefore, the following comparisons were made only between the BM and the FOM (the last one is the reference model). FOMs’ and BMs’ standardized factor loadings and FOMs’ factor correlations can be seen in **Figures 2**, **3**. The BMs common dimension (i.e., global evaluation) showed lower loadings when compared with FOMs’ loadings, as where the variance was split into a greater number of components. The estimated factor loadings in the common dimension in the three samples were moderate to high. When comparing the samples, we found slightly lower factor loadings for the items in Sample_G_ (*M* = 0.57, range = [0.29, 0.73]) and in Sample_D_ (*M* = 0.58, range = [0.38, 0.78]) than in Sample_F_ (*M* = 0.65, range = [0.46, 0.79]).

In the case of the specific dimensions of competence, morality, and sociability, the magnitude of the estimated factor loadings changed considerably by sample. For the competence dimension, the unsigned average factor loading was equal to 0.46 (range = [0.39, 0.56]) for Sample_G_, 0.26 (range = [-0.11, 0.55]) for Sample_F_, and 0.71 (range = [0.67, 0.74]) for Sample_D_; for the morality dimension, the unsigned average factor loading was equal to 0.52 (range = [0.39, 0.58]) for Sample_G_, 0.41 (range = [0.02, 0.61]) for Sample_F_, and 0.25 (range = [-0.53, 0.31]) for Sample_D_; and for the sociability dimension, the average factor loading was equal to 0.35 (range = [0.20, 0.63]) for Sample_G_, 0.41 (range = [0.31, 0.56]) for Sample_F_, and 0.46 (range = [0.24, 0.61]) for Sample_D_.

In Sample_G_ (and in Sample_F_ and Sample_D_) the BM’s general dimension (i.e., global evaluation) accounted for 60% (78 and 57%) of the common variance. By contrast, the specific dimensions were able to explain only a considerably lower percentage of the common variance: 11% (6 and 27%) in the competence dimension, 20% (6 and 6%) in the morality dimension, and 9% (9 and 10%) in the sociability dimension. Latent factor variables were not statistically different from 0 in the sociability dimension in Sample_G_, in competence and morality dimensions in Sample_F_, nor in the morality dimension in Sample_D_. The variability in the distribution of variance among general/specific dimensions and samples, offers interesting results. On one hand, it may be affirmed that the Sample_F_ item responses were the most influenced by the global dimension. On the other hand, the weight of the morality specific dimension in Sample_G_ was remarkable in contrast to the other two samples. Finally, in Sample_D,_ the competence specific dimension was, among the specific dimensions, the one with the most percentage of variance due to its specific content (in general and in contrast to the other two samples).

### Estimation of Reliability

Cronbach’s alpha, omega and hierarchical omega were estimated for the BM and the FOMs of competence, morality, and sociability along with the SDE in the three samples (**Table 3**). The Cronbach’s alpha and omega estimations (including the SDE) were high enough in the three samples. Coefficient omega hierarchical estimations for the reliability of the specific dimensions beyond the variance accounted for the general factor were notably low for all specific factors except in the competence dimension in Sample_D_ (its omega hierarchical estimation was under the limit to be considered acceptable but even so it was significantly higher than the rest). On the other hand, coefficient omega hierarchical estimations were high enough for the global evaluation factor in the three samples. According to hierarchical omega estimations, the only dimension that can be assessed using observable variables with sufficient reliability is the common dimension (i.e., the global evaluation).

**Table 3:** Reliability, Omega and Hierarchical Omega for three subdimension models in the three samples.

	Cronbach’s alpha			Omega			Hierarchical Omega		
			
	Sample_G_	Sample_F_	Sample_D_	Sample_G_	Sample_F_	Sample_D_	Sample_G_	Sample_F_	Sample_D_
**Stereotype dimensions**									
Global evaluation	0.89	0.91	0.89	0.91	0.92	0.91	0.75	0.87	0.77
Competence	0.78	0.73	0.88	0.79	0.73	0.89	0.35	0.11	0.66
Morality	0.89	0.85	0.84	0.89	0.85	0.84	0.38	0.06	0.00
Sociability	0.78	0.78	0.74	0.79	0.79	0.76	0.22	0.26	0.34
**SDE**	0.80	0.77	0.74	0.80	0.78	0.75			


**SDE*, Semantic Differential of Evaluation.*

### Evidence of Validity Based on Relationships with Measures of Other Variables

**FIGURE 4:**
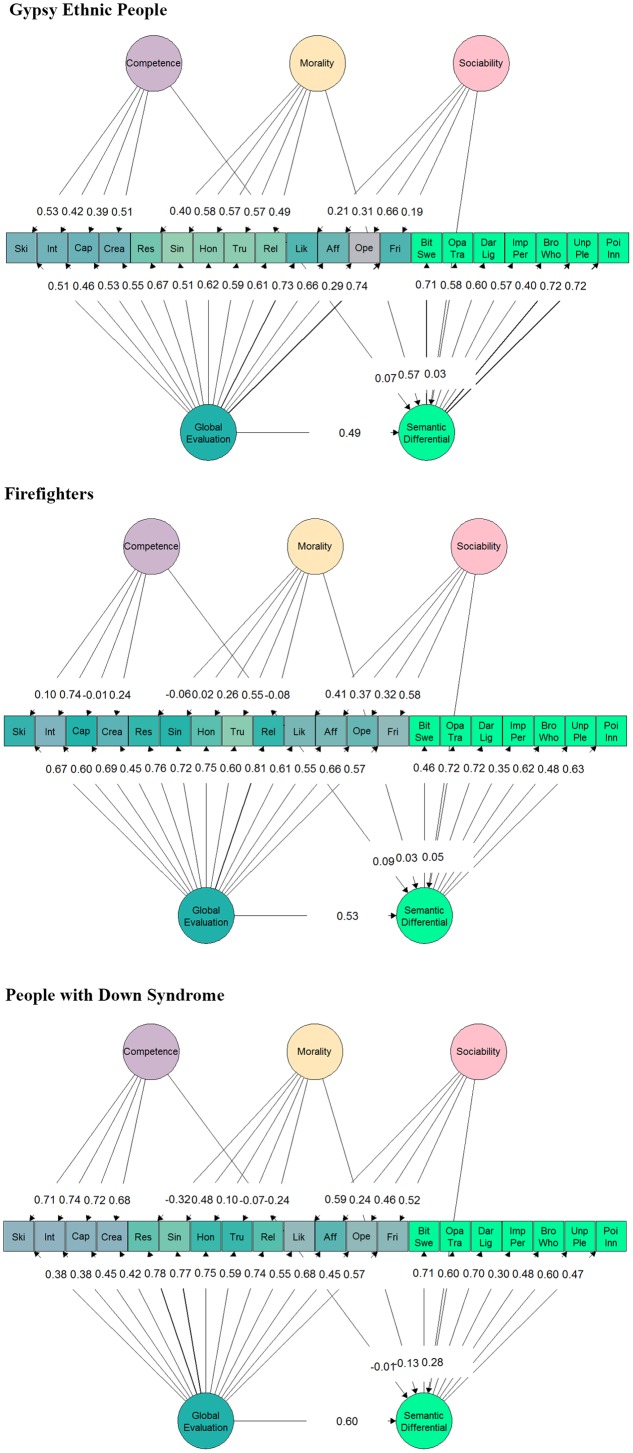
Standardized factor loadings in Bi-factor model with three subdimensions (BM) in the three samples. Latent variable colors determine, based on their relationship, the color of the manifest variables. All factor loadings of the global factor are statistically significant. In the case of the specific factors, items loadings in all the Competence and Morality items in Sample_B_, and honest and trustworthy in Sample_D_ are not statistically significant. All global evaluation regression coefficients are statistically significant. In the case of the specific factors only regression coefficients for Morality in Sample_G_ and sociability in Sample_D_ are statistically significant.

The fit indicators of the models that related BM with SDE in the three samples are shown in the **Table 2** and standardized loadings and regression coefficients can be seen in **Figure 4**. The chi-squared test showed lack of fit with data in all models. The opposite happened with other less strict indicators of fit. In the three samples, the fit indicators were acceptable for the BM with the SDE. The standardized regression coefficients for the common dimension were not as high as we expected, as only moderate statistically significant correlations were found in Sample_G_ (0.49, *p* < 0.001), in Sample_F_ (0.53, *p* < 0.001), and in Sample_D_ (0.60, *p* < 0.001). The standardized regression coefficients for the specific dimensions (i.e., competence, morality, and sociability) were statistically significant only between morality and the SDE in Sample_G_ (0.57, *p* < 0.001) and between sociability and the SDE in Sample_D_ (0.28, *p* < 0.001).

## Discussion

The aim of this work was to explore whether there was a better and more useful way to explain the shared variance among competence, morality, and sociability dimensions. BMs have been proposed as an alternative to the commonly used FOMs. BMs are an option to identify the shared variance among competence, morality, and sociability dimensions (or competence and warmth dimension subsuming morality and sociability) due to a common dimension of global evaluation toward the perceived group. Moreover, BMs are able to guarantee access to the specific content of the morality, competence and sociability dimensions depending on the group. This internal structure, instead of wasting the utility of employing the shared variance, is able to identify the shared variance as a latent variable (the global evaluation), which influences (to a greater or lesser degree) all the answers to stereotype items.

### Bi-factorial Model vs. Alternative Models to Measure the Stereotype Content

The results obtained by this study revealed that two sources of variance account better the variance of the items of competence, morality, and sociability dimensions. In addition, the findings indicated that when a group is perceived the main source of variance in all items of stereotype content might be due to global evaluation (valence and intensity) of the group. Fit differences between BMs and FOMs (the most common in the literature) demonstrated that BMs had a better fit. Specifically, BMs statistically and practically produced better results than FOMs in the three samples. On the other hand, single factor models (SFMs) representing only a global evaluation have resulted to be the worst option for exploring stereotype content as they cannot account for the variance due to the specific content of the stereotype dimensions. Therefore, it may be assumed that the stereotype content reflects more than merely the content linked to the competence, morality, and sociability dimensions (and more than only a global evaluation). This assumption matches the proposed initial hypothesis, in concordance with the halo effect described by Judd et al. (2005). However, we have reasons to assume that this big amount of variance is more than a mere halo effect. If correlations among stereotype content dimensions were only due to a halo effect this effect would also influence negative dimensions such as immorality. Nevertheless, Sayans-Jiménez et al. (2017) have shown that immorality is negatively related to the rest of stereotype content. For this reason, we consider that the evaluative component of the stereotype content is real and it is indeed expressing the evaluation toward the perceived group. In addition, it is important to highlight that the results were replicated in three samples with three different outgroups and with three different patterns of response (i.e., high/low and ambivalent stereotypes).

The fact that there is a shared dimension between all the items, explaining between 57 to 78% of the common variance, can discourage any inferences referring to the specific content of the stereotype dimensions. However, this does not mean that the competence, morality, and sociability dimensions (or competence and warmth dimension subsuming morality and sociability) cannot be measured. This means only that, in the presence of high correlations among the mentioned stereotype dimensions, it is necessary to partial out the variance not related with the specific content of each dimension (once this common variance is partialled out it is possible that a higher number of items in each dimension will be required to reach satisfactory reliability estimations). As it can be seen in our results, the chance to measure the specific stereotype content depends on the group under evaluation. In some groups (probably with fewer specific available information) only global evaluations should be performed, whereas in other groups it would be possible to access to a more specific variance related to competence, morality, and sociability. Furthermore, evidence of validity based on relationships with measures of other variables supports that this large amount of shared variance is due to the global evaluation made of the outgroup target. Therefore, considering that the theoretical model (Fiske et al., 2002) do not take into account the effect of such correlations among the mentioned stereotype dimensions, this leads to the waste of a large amount of information related to the global evaluation of groups (or people belonging to them).

Attending only to the special case of the ambivalent outgroup (Sample_D_), the BM has demonstrated a greater potential to capture enough variance with the competence specific dimension. The higher probability of non-related variations between the specific dimensions associated with ambivalent outgroups (that is why they are called ambivalent) would contribute to influencing the raters to make greater distinctions when they answer to the attributes of each dimension. However, this effect was still not enough to make possible the measuring of the specific content of competence with observable variables with adequate reliability estimations.

### Utility of Bi-factorial Model to Access to Specific Content of the Stereotype Dimensions

The results of this work support the use of BMs against the FOMs commonly used (Fiske et al., 1999; Eckes, 2002; Lin et al., 2005; López-Rodríguez et al., 2013), both with high/low-valued outgroups and with ambivalent-valued outgroups. In addition, these results endorse the BMs as an alternative to the second order models proposed by Srivastava et al. (2010). Although Srivastava et al. (2010) studied the basic dimensions for interpersonal perception, the way in which these dimensions are structured may be similar to the approach adopted by BMs (for a detailed analysis regarding the relation between agency and communion and the stereotype dimensions used in this study, see Wojciszke and Abele, 2008 and Brambilla and Leach, 2014).

BMs have utility for interpreting stereotype content, allowing inferences to be raised with respect to both global and specific factors: (1) The global evaluation (positive/negative), which is influenced by the connotative value of the target’s social category belonging (i.e., it is possible to locate the outgroup in a positive/negative continuum), and (2) the specific content of competence, morality, and sociability dimensions, which is related in a greater extent to more reflexive or reasoned evaluations. For this reason, BMs are considered to be more amply supported by empirical evidence than the FOMs or other alternative models currently used.

The significant weight of the global evaluation component in the stereotype content may be related to the importance of the morality dimension when social objects are evaluated. In fact, the semantic definition of both dimensions (global evaluation and morality) could be the same; and morality items are the main contributors of the global evaluation variance. The content of the morality dimension could be decisive to provide the valence of the global evaluations (Wojciszke et al., 1998). Van Bavel et al. (2012a) highlight the importance of the moral content: When people have moral awareness (something related to social object perception), the target construal is influenced by moral intuitions that will determine the evaluative process (Van Bavel et al., 2012a). This will result in faster evaluations, which are also more extreme and more associated with universal prescriptions (i.e., everybody should or should not behave in a specific way according to each situation; Van Bavel et al., 2012a). The importance of the content related to morality is widely supported (Brambilla and Leach, 2014; Leach et al., 2015) and we think that it is univocally connected to the social target evaluation. This content is related to the way in which people evaluate other groups and persons belonging to them and it can explain why morality is also related to other psychosocial processes (vs. the content of competence and sociability) such as acculturation, perceived threat, identity, realistic competition (Brambilla et al., 2012, 2013; Goodwin et al., 2014; Kervyn et al., 2015; López-Rodríguez and Zagefka, 2015).

On the other hand, the greater influence of the global evaluation may be due to other factors. Dominant among these are: lack of concretion of the attributes employed in the measurement; the fact that the target evaluated belongs to vague and diffuse categories; the raters’ lack of information regarding the evaluated object (e.g., the raters can only evaluate a small sample of rated behaviors); the raters’ perception that the categories covary with general impressions; and the raters’ lack of effort or the raters’ cognitive distortions (Cooper, 1981; Feeley, 2002). However, it must be said that, although the information on the global evaluation is of key importance, the specific content of morality, competence and sociability are still of great importance, both in the field of impression formation (Goodwin et al., 2014) and in the prediction of intergroup emotional reactions and behaviors (Cuddy et al., 2008). Therefore, more studies are required to reduce the amount of shared variance among competence, morality and sociability.

In the case of the results of this study, some of the reasons highlighted above may be related to differences in the respective and relative weighting of the global evaluation and specific dimensions within the total variance (i.e., the percentage of common variance accounted for by the global dimension). Features such as entitativity or essentialism of a group can accentuate the process of social categorization (Yzerbyt and Demoulin, 2010). It is likely that social categorization was stronger for the people of the gypsy ethnic group or with Down syndrome compared to professional firefighters the firsts two ones are outgroups with a shared culture and/or with a greater perceived physical homogeneity. Secondly, more controlled and with more specific information answers are expected from samples of people in the gypsy ethnic group and with Down syndrome as the targets due to the influence of social desirability. The features of both groups may have contributed to reducing the effect of global evaluation of Sample_G_ and Sample_D_.

Future studies should determine whether the specific low variance of the specific dimensions is attributable, not only to any of the factors mentioned above, but to the level of specificity at which the stereotypes have been measured (i.e., decontextualized group in contrast to a well-defined interaction including context and results); or if on the contrary a “pure” measurement of these contents cannot be performed. Finally, although it was not one of the goals of this study, the results of this research have shown that the measurement models that split warmth into morality and sociability have shown an adequate fit in accordance with what was theoretically expected (Leach et al., 2007, 2015; Brambilla and Leach, 2014; Goodwin et al., 2014).

## Conclusion

This study has highlighted that the responses to the items aimed at measuring the stereotype content may share a common overall evaluative factor with respect to the attitude object, which could be very useful in predicting intergroup behaviors in the case of stereotypes. In addition, the need to control the variance due to this common factor or to improve access to pure content of the dimensions of competence, morality, and sociability (or competence and warmth dimension subsuming morality and sociability) is highlighted. This is the recommend for predicting specific emotional responses to the position in each of the dimensions of the evaluated social object.

The BMs allow obtaining intergroup global evaluations through the stereotype content in a straight and simple way. It is necessary to test whether the use of these models can address, for example, the study of ambivalent evaluations toward outgroup targets expressed through the answers to self-report of emotional reactions (e.g., it may be possible to split the evaluative content of envy or compassion and access their specific content, if it is available). It is also necessary to consider whether BMs may be potentially applicable to structural models that relate the stereotype content with emotional reactions and behavioral tendencies. Finally, given the theoretical reasons for applying BMs, it is of great importance to check the relationship between the results found with this type of model and those obtained with techniques that explore the generation of automatic or implicit evaluations.

## Author Contributions

PS-J developed the theoretical framework, designed the study, conducted most of the data analyses, and wrote the majority of the text. IC, AR, and JB assisted and advised with the theoretical framing, constructed variables, and assisted with data analyses.

## Conflict of Interest Statement

The authors declare that the research was conducted in the absence of any commercial or financial relationships that could be construed as a potential conflict of interest.

## Footnotes

1 There are studies in Spain that show how the gypsy ethnic people are worse evaluated than Spaniards in general (see Gómez, 2009; Navas and Cuadrado, 2012).

2 In the Appendix 2 it can be seen the measurement models for Competence, Morality, Sociability, and SDE. Reliability estimation of these models is equivalent to those reliability estimations performed on FOMs and they can be seen in **Table 3**.

3 This model was also tested with two stereotype content dimensions (morality and sociability items loaded in the warmth dimension, FOM_2_). Although empirical evidence supporting the three-dimension model has been presented in previous studies (Leach et al., 2007; Brambilla et al., 2011, 2012; Brambilla and Leach, 2014; Goodwin et al., 2014), the two-dimension model was included in the Appendix 3 (together with fit results of all the models) due to its common use and to provide more evidence supporting the division of warmth into morality and sociability.

4 This second model was also tested both with two specific dimensions (competence and warmth—BM_2_) and with three specific dimensions (competence, morality, and sociability). In order to save space and to reduce the complexity of this section their fit indicators are shown in the Appendix 3.
