# Supplementary material for: Extracting the Evaluations of Stereotypes: Bi-factor Model of the Stereotype Content Structure
Source: Front Psychol. 2017 Oct 4;8:1692. doi: 10.3389/fpsyg.2017.01692 (PMC5649216; doi:10.3389/fpsyg.2017.01692)
Supplement: Supplementary file 1 [file Appendix_1.docx]

**Appendix 1**

**Instructions for the measurement of the intergroup attitude toward gypsy ethnic group (or professional firefighters or people with Down syndrome)**

Now we are going to ask a few questions to assess your ability to perceive certain characteristics in non-familiar people of the gypsy ethnic group (or professional firefighters group or people in the Down syndrome group).

In this task you have to imagine a large group of people who represent all types of people you think exist in the gypsy ethnic group (or professional firefighters group or people with Down syndrome group). Imagine, for example, a group of over 300 non-familiar people of the gypsy ethic group (or professional firefighters group or people in the Down syndrome group).

Take your time and answer the following questions imagining a large group of people who represent all types of people in the gypsy ethnic group (or professional firefighters group or people in the Down syndrome group).

Try to guess about how many people in this group of non-familiar people in the gypsy ethnic group (or professional firefighters group or people with Down syndrome group), representing all types of people in this group, have the qualities displayed below.
None /Almost none/Few/Half/Many/Most/All**Instructions for the measurement of the Semantic Differential toward gypsy ethnic group (or professional firefighters or people with Down syndrome)**

**Semantic Differential instructions**

The following task challenges you, as “not gypsy” (nor professional firefighter nor people with Down syndrome), to say to what extent certain characteristics are associated with people of the gypsy ethnic group (or professional firefighters group or people with Down syndrome) whom you do not know at all. For this, pairs of characteristics (e.g., dangerous-safe) will be given and you must answer, as fast as you can, to what extent you relate people of the gypsy ethnic group (or professional firefighters group or people with Down syndrome group), whom you do not know at all, to the characteristics presented below. The closer you make the cross to one characteristic or the other, the closer the relationship you are expressing between the characteristic and people in the gypsy ethnic group (or professional firefighters group or people with Down syndrome group) whom you do not know at all. For example, in the case of the dangerous-safe characteristics, you might choose *very dangerous, rather dangerous, slightly dangerous, partly dangerous, partly safe, slightly safe, rather safe, and very safe.*

Although it may be difficult to find the relationship for some of the characteristics in each pair, please try to answer all the pairs of characteristics as fast as possible.
