# Supplementary material for: Extracting the Evaluations of Stereotypes: Bi-factor Model of the Stereotype Content Structure
Source: Front Psychol. 2017 Oct 4;8:1692. doi: 10.3389/fpsyg.2017.01692 (PMC5649216; doi:10.3389/fpsyg.2017.01692)
Supplement: Supplementary file 2 [file Appendix_2.docx]

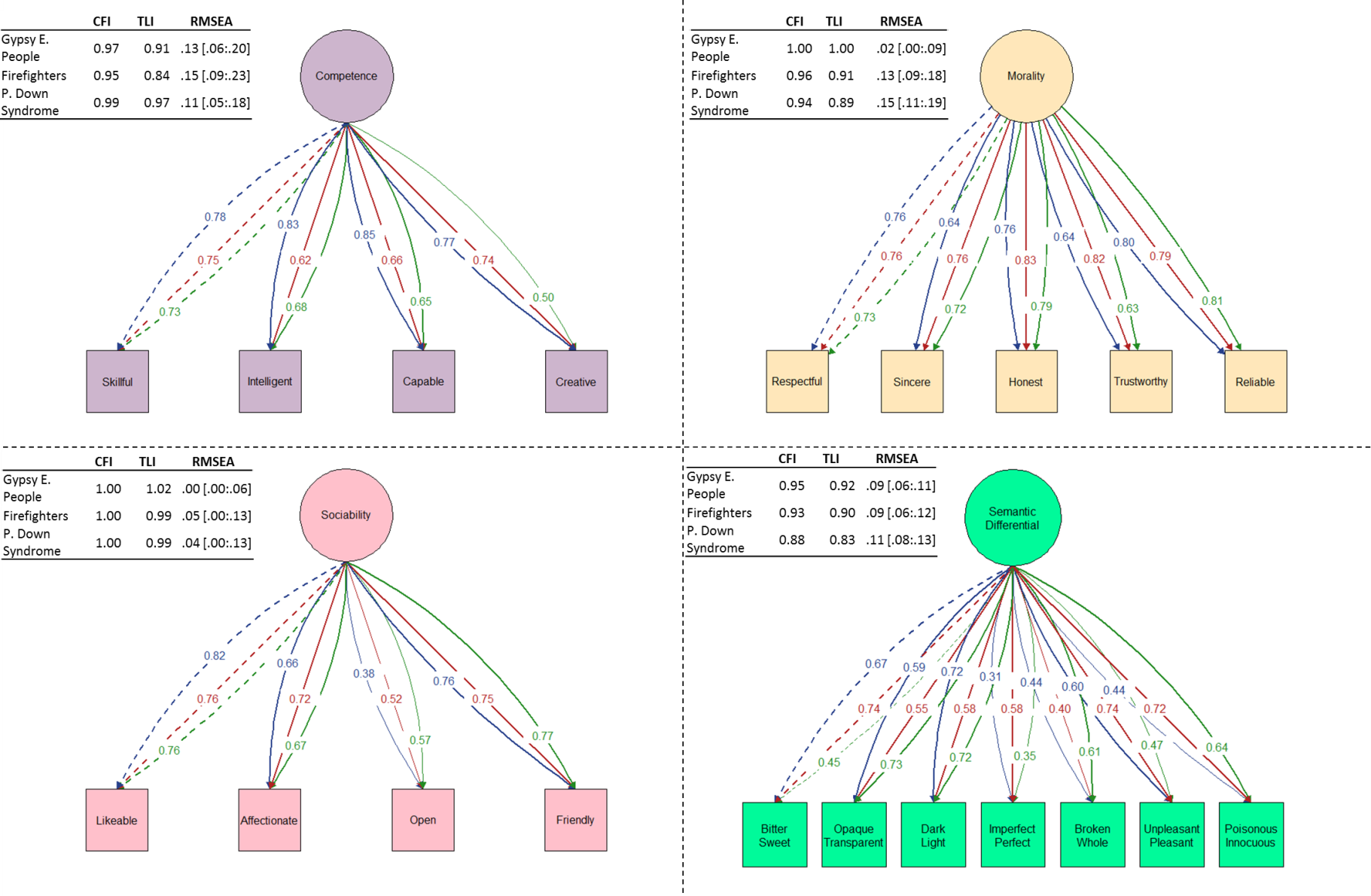


**Appendix 2**

Measurement models for each latent variable in each group separately. Dashed edges represent *a priori* fixed factor loading. Edge and standardized factor loadings colors express the information of each outgroup, blue for gypsy ethnic people, red for firefighters, and green for people with Down syndrome. Fit indicators of each model are shown in the upper left corner of each model.
