# Supplementary material for: Extracting the Evaluations of Stereotypes: Bi-factor Model of the Stereotype Content Structure
Source: Front Psychol. 2017 Oct 4;8:1692. doi: 10.3389/fpsyg.2017.01692 (PMC5649216; doi:10.3389/fpsyg.2017.01692)
Supplement: Supplementary file 3 [file Appendix_3.docx]

**Appendix 3**

| *Fit statistics, in all the samples, for Single Factor Model (SFM), First Order Model with two subdimensions (FOM_2_), First Order Model with three subdimensions (FOM_3_), Bi–factor Model with two subdimensions (BM_2_), Bi–factor Model with three subdimensions (BM_3_), and BM_3_ related with Semantic Differential of Evaluation (BM_3_ & SDE)* | | | | | | | | | | |
| --- | --- | --- | --- | --- | --- | --- | --- | --- | --- | --- |
|  | | χ2 | ∆ χ2 | Df | ∆Df | RMSEA [90% CI] | CFI | ∆CFI | TLI |  |
| **Model comparison** | |  |  |  |  |  |  |  |  |  |
| Sample_G_ | SFM | 437.52 | 319.44** | 65 | 3 | .14 [.13, .15] | .79 | –.18 | .75 |  |
|  | FOM_2_ | 285.68 | 167.6** | 64 | 2 | .11 [.10, .12] | .87 | –.10 | .85 |  |
|  | FOM_3_ | 118.08 | .00 | 62 |  | .06 [.04, .07] | .97 | .00 | .96 |  |
|  | BM_2_ | 84.57 | –33.51** | 52 | –10 | .05 [.03, .06] | .98 | .01 | .97 |  |
|  | BM_3_ | 91.00 | –27.08* | 52 | –10 | .05 [.03, .07] | .98 | .01 | .97 |  |
| Sample_F_ | SFM | 248.78 | 80.67** | 65 | 3 | .10 [.08, .11] | .90 | –.04 | .88 |  |
|  | FOM_2_ | 241.24 | 73.13** | 64 | 2 | .10 [.08, .11] | .90 | –.04 | .89 |  |
|  | FOM_3_ | 168.11 | .00 | 62 |  | .08 [.06, .09] | .94 | .00 | .93 |  |
|  | BM_2_ | 141.64 | –26.47* | 52 | –10 | .08 [.06, .09] | .95 | .01 | .92 |  |
|  | BM_3_ | 125.27 | –42.84** | 52 | –10 | .07 [.05, .08] | .96 | .02 | .94 |  |
| Sample_D_ | SFM | 733.34 | 511.58** | 65 | 3 | .18 [.17, .19] | .67 | –.25 | .60 |  |
|  | FOM_2_ | 303.18 | 81.42** | 64 | 2 | .11 [.10, .12] | .88 | –.04 | .86 |  |
|  | FOM_3_ | 221.76 | .00 | 62 |  | .08 [.08, .10] | .92 | .00 | .91 |  |
|  | BM_2_ | 170.73 | –51.03** | 52 | –10 | .09 [.07, .10] | .94 | .02 | .91 |  |
|  | BM_3_ | 150.66 | –71.1** | 52 | –10 | .08 [.06, .09] | .95 | .03 | .93 |  |
| **Structural models** | |  |  |  |  |  |  |  |  |  |
| Sample_G_ | BM_3_ & SDE | 290.80 |  | 153 |  | .06 [.05, .06] | .95 |  | .93 |  |
| Sample_F_ | BM_3_ & SDE | 317.35 |  | 153 |  | .06 [.05, .07] | .93 |  | .91 |  |
| Sample_D_ | BM_3_ & SDE | 395.29 |  | 153 | 3 | .07 [.06, .08] | .91 |  | .89 |  |
| *Note.* χ2 = Chi–square test; ∆χ2 = statistically significant Chi–square differences at *p* < .001 by sample between the model and the FOM; Df = Degrees of freedom; ∆Df = Degrees of freedom differences by sample between the model and the FOM; RMSEA = Root Mean Square Error of Approximation; 90% CI = 90% Confidence Interval of the RMSEA; CFI = Comparative Fit Index; ∆CFI = CFI differences by sample between the model and the FOM_3_; TLI = Tucker–Lewis Index.  **p* < .01, ***p* < .001. | | | | | | | | | | |
